# Development and measurement properties of the AxEL (attitude toward education and advice for low-back-pain) questionnaire

**DOI:** 10.1186/s12955-021-01908-4

**Published:** 2022-01-10

**Authors:** Edel T. O’Hagan, Ian W. Skinner, Matthew D. Jones, Emma L. Karran, Adrian C. Traeger, Aidan G. Cashin, Benedict M. Wand, Siobhan M. Schabrun, Sean O’Neill, Ian A. Harris, James H. McAuley

**Affiliations:** 1 Centre for Pain IMPACT, Neuroscience Research Australia (NeuRA), 139 Barker Street, Randwick, NSW 2031 Australia; 2 Prince of Wales Clinical School, University of New South Wales, Sydney, NSW Australia; 3 School of Community Health, Charles Sturt University, Port Macquarie, NSW Australia; 4 School of Health Sciences, Faculty of Medicine and Health, University of New South Wales, Sydney, NSW Australia; 5 IIMPACT in Health, UniSA: Allied Health and Human Performance, The University of South Australia, Adelaide, SA Australia; 6 Institute for Musculoskeletal Health, Sydney School of Public Health, Faculty of Medicine and Health, The University of Sydney, Sydney, NSW Australia; 7 Faculty of Medicine, Nursing and Midwifery and Health Sciences, The University of Notre Dame Australia, Fremantle, WA Australia; 8 South Western Sydney Clinical School, Liverpool Hospital, The University of New South Wales, Sydney, NSW Australia; 9 Institute of Bone and Joint Research, Kolling Institute, The University of Sydney, Sydney, NSW Australia; 10 Ingham Institute of Applied Medical Research, Liverpool, Sydney, NSW Australia

**Keywords:** Low back pain, Questionnaire development, Measurement properties, First-line care

## Abstract

**Introduction:**

Clinician time and resources may be underutilised if the treatment they offer does not match patient expectations and attitudes. We developed a questionnaire (AxEL-Q) to guide clinicians toward elements of first-line care that are pertinent to their patients with low back pain.

**Methods:**

We used guidance from the COSMIN consortium to develop the questionnaire and evaluated it in a sample of people with low back pain of any duration. Participants were recruited from the community, were over 18 years and fluent in English. Statements that represented first-line care were identified. Semantic scales were used to measure attitude towards these statements. These items were combined to develop the questionnaire draft. Construct validity was evaluated with exploratory factor analysis and hypotheses testing, comparing to the Back Beliefs Questionnaire and modified Pain Self-Efficacy Questionnaire. Reliability was evaluated and floor and ceiling effects calculated.

**Results:**

We recruited 345 participants, and had complete data for analysis for 313 participants. The questionnaire draft was reduced to a 3-Factor questionnaire through exploratory factor analysis. Factor 1 comprised 9 items and evaluated *Attitude toward staying active*, Factor 2 comprised 4 items and evaluated *Attitude toward low back pain being rarely caused by a serious health problem,* Factor 3 comprised 4 items and evaluated *Attitude toward not needing to know the cause of back pain to manage it effectively*. There was a strong inverse association between each factor and the Back Beliefs Questionnaire and a moderate positive association with the modified Pain Self-Efficacy Questionnaire. Each independent factor demonstrated acceptable internal consistency; Cronbach α Factor 1 = 0.92, Factor 2 = 0.91, Factor 3 = 0.90 and adequate interclass correlation coefficients; Factor 1 = 0.71, Factor 2 = 0.73, Factor 3 = 0.79.

**Conclusion:**

This study demonstrates acceptable construct validity and reliability of the AxEL-Q, providing clinicians with an insight into the likelihood of patients following first-line care at the outset.

**Supplementary Information:**

The online version contains supplementary material available at 10.1186/s12955-021-01908-4.

## Introduction

Low back pain is the leading contributor to the global disability burden [1]. Between 1990 and 2019, low back pain caused one of the largest absolute increases in the number of days lost to disability of any health condition, and this upward trajectory is predicted to continue, exacerbating demands on health systems [2]. Clinical practice guidelines for the management of low back pain consistently recommend that first-line care should include advice, education and reassurance to minimise unnecessary interventions and decrease the burden on health systems [3, 4]. Regardless of the duration of low back pain, clinicians should provide advice to remain active, education on the benign nature of low back pain, and reassurance about the absence of a serious medical condition [5–7].

Systematic reviews have highlighted the gap between clinical practice guideline recommendations for first-line care and the care that is usually provided to people with low back pain [8, 9]. The suboptimal use of first-line care is influenced by both clinician and patient-related factors [10]. For example, clinicians often report insufficient time and resources to appropriately provide first-line care within the time constraints of an initial consultation [11]. For patients, adherence to first-line care is influenced by their expectations for treatment and attitudes towards the specific health care behaviour [12, 13]. A greater understanding of patient attitudes toward first-line care could assist clinicians in providing more efficient and effective consultations within the time constraints of clinical practice. Clinicians could personalise the care provided to ensure that it aligns with the patient’s attitudes and expectations which may improve patient satisfaction, better facilitate delivery of first-line care, and potentially improve treatment outcomes [10].

A questionnaire to assess patient attitudes toward first-line care for low back pain could provide clinicians with valuable insight to indicate which components of first-line care people with low back pain are likely to engage with. Questionnaires to measure attitude in people with pain are available but have notable shortcomings. Common limitations include only measuring one component of attitude and having a sizable participant burden. For example, the Pain and Impairment Relationship Scale assesses beliefs and attitudes associated with the experience of chronic pain and one’s ability to function despite pain. Attitude and beliefs are measured using a series of 15 agree/disagree questions only [14]. Similarly, the Survey of Pain Attitudes questionnaire assesses 7 pain-related beliefs. Participants indicate their agreement level with 57 statements about low back pain on a 5-point Likert scale, ranging from true or false, with intermediate labels, possibly false, unsure, possibly true [15]. The Back Pain Attitude Questionnaire also requires participants to rate their agreement level with statements on a 5-point Likert scale [16]. Evaluation of the measurement properties suggest that each of these questionnaires demonstrate adequate internal consistency, test re-test stability and hypothesis testing support the convergent and discriminant validity [14–18]. Despite the individual merit of each of these questionnaires, currently there is no reliable method to understand patient attitudes toward first-line care for low back pain, and so valuable clinician time and resources may be underutilised or misdirected when providing first-line care.

Recently a panel of expert researchers and clinicians joined with consumers to compile a list of evidence-based “*essential key messages”* that the public should know about low back pain [19]. The resulting list included messages about first-line care. We developed a questionnaire to measure attitudes toward those messages that could help clinicians tailor their advice to patients. For example, a negative attitude toward a message of education on the benign nature of low back pain could lead a clinician to discuss that message. Whereas a positive attitude toward a message about advice to stay active would indicate to the clinician may not have to spend time re-enforcing that message. To be useful such a tool should demonstrate reliability and construct validity.

This study aimed to.

(i) Develop a new patient-reported questionnaire, the Attitude toward Education and advice for Low back pain-Questionnaire (AxEL-Q).
(ii) Evaluate the construct validity and reliability of the AxEL-Q

## Methods

**Fig. 1:**
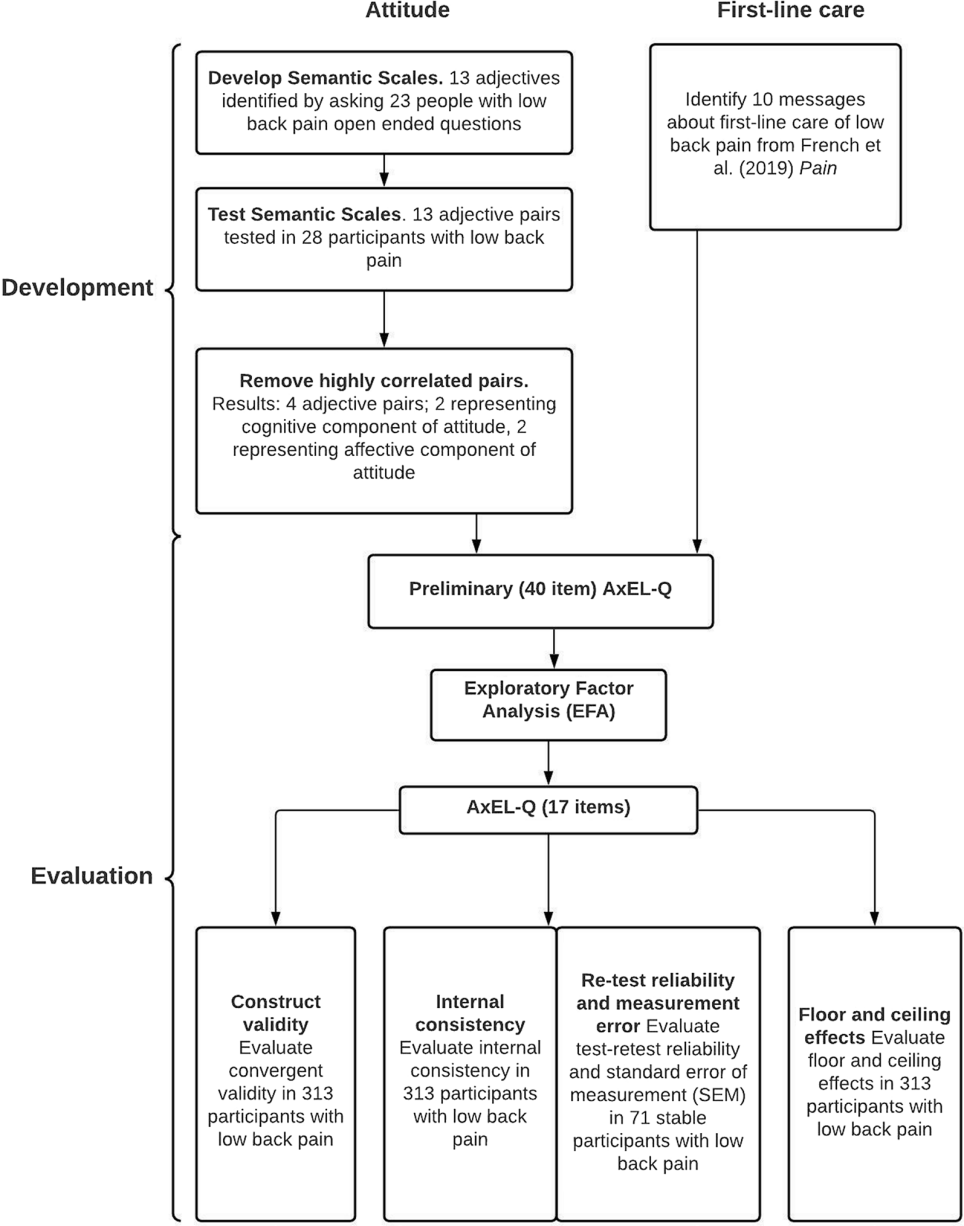
Development and evaluation of the AxEL-Q

This study describes the development and evaluation of a questionnaire to assess patient attitudes toward first-line care for low back pain. The process is outlined in Fig. 1. We used the COSMIN consortium developed study design checklist [20, 21], guidelines on terminology [22], and risk of bias checklist for clinimetric studies [23] in designing and reporting this study. This study was approved by the University of New South Wales Human Research Ethics Committee prior to data collection (approval number 17919). The protocol was pre-registered on the Open Science Framework in August 2018. (https://osf.io/9wncz/).

### Conceptual framework

The AxEL-Q is based on a reflective model where all items are presumed to measure the same underlying construct, i.e., attitude toward first-line care for low back pain. Attitude toward first-line care for low back pain is a multi-dimensional construct measured indirectly using multiple observable items. The corresponding measurement theory is Classical Test Theory where the observed score of an item is the ‘true’ score of the construct to be measured *plus* the error term for that item [24].

### Development of the preliminary AxEL-Q

#### Source population

We developed the preliminary questionnaire in two independent samples of people with low back pain. Participants were recruited consecutively from databases of people who expressed an interest in research. Participants had low back pain of any duration; all were in Australia and fluent in English. Participants ranged in age from 18 to 70 years.

#### Item identification

##### Attitude

Attitude comprises affective components relating to feelings and emotions and cognitive components relating to thoughts and beliefs [25]. Measuring attitude should incorporate both affective and cognitive components. The affective component is evaluative, and because attitudes are always evaluating an object*,* there is inevitably a cognitive component to represent the object of thought [26]. We used semantic differential scales, which are considered appropriate to measure attitude [27]. Semantic differential scales measure responses in terms of ratings on bipolar scales defined with contrasting adjectives at each end [28].

##### First-line care

A Delphi study [19] identified 30 messages considered important for the public to know about diagnosis, imaging, and self-care for people with low back pain. These 30 messages covered six themes; red flag identification, disease knowledge, reassurance, stay active, unnecessary interventions and principles of management [19]. The messages were compiled to inform education resources, including new websites and other educational material [19]. Through discussion, four research team members (EO, JM, IH, BW) selected ten messages that best represented first-line care [29]. The messages included at least one message from each of the six themes identified in the Delphi study to ensure comprehensiveness and comprehensibility. These messages were inherently relevant to the public because that was an important target of the Delphi study [19].

#### Procedure

To identify the anchors for the semantic differential scales, we conducted face-to-face interviews and online questionnaires. We presented the first independent sample of participants with low back pain (n = 23) with 10 messages that represented first-line care, and in a face-to-face interview, asked them two questions; “as someone with low back pain, what word would you use to describe this message” and “how does this message make you feel?” These open-ended questions generated 13 unique adjectives; (depressing, understanding, sad, confusing, anxious, interesting, discouraged, helpful, well cared for, agree, worried, frustrated, surprised). This method accounted for the anchors being relevant and comprehensible to the target population. One researcher (EO) contributed both complementary (e.g., a straightforward negative such as supportive/unsupportive) and graded (e.g., reassuring/confusing) antonyms to provide a second anchor and create thirteen semantic scales. A description of the process to develop the semantic differential scales is available in Additional file 1.

#### Item reduction

We tested the semantic differential scales in the second independent group of participants with low back pain (n = 28). We constructed a questionnaire in a secure electronic form using Qualtrics [30]. This questionnaire presented 10 messages representing first-line care and asked participants to rate their responses using the thirteen semantic differential scales. We measured the correlation (Pearson’s r) between items. We set the multicollinearity threshold at r > 0.85, identified highly correlated pairs and eliminated one pair through discussion. The removed adjective pairs included depressing-hopeful, irrational-understandable, sad-happy, confusing-informative, anxious-confident, boring-interesting, discouraged-motivated, unhelpful-helpful, not cared for-well cared for. We chose two adjective pairs to represent affective components of attitude; frustrated-encouraged, worried-reassured, and two adjective pairs to represent cognitive components of attitude; disagree-agree, surprising-expected. Full details are available in Additional file 1.

##### Expert focus group

An expert focus group (n = 9) reviewed the final adjective pairs. The expert focus group consisted of five researchers and four clinicians with an interest in musculoskeletal health. The focus group included researchers with a range of experience from a graduate to senior researchers with over 20 years of research experience; the clinicians were all senior clinicians, each with over 15 years of clinical experience. The expert focus group did not recommend any changes to the semantic scales. We named the questionnaire the “AxEL-Q”; (**A**ttitude toward Education and advice for Low-back-pain) Questionnaire.

#### Questionnaire draft

The resulting preliminary AxEL-Q questionnaire had 40 items that included questions about the affective and cognitive components of attitude relative to ten statements of education and advice to represent first-line care. Each item was scored on a 0–6 Likert scale. The full details of the structure of the preliminary AxEL-Q, including details on the instructions given, response options and scoring algorithm, are available in Additional file 2.

## Evaluation of the AxEL-Q

### Participants

We evaluated the questionnaire draft in a sample of people with low back pain. We recruited people who had low back pain of any duration. This was a global study, there were no restrictions on what country or setting people were recruited from. Study participants were required to be aged over 18-years and fluent in English. We recruited one sample to represent the target population regarding age, sex, and important disease characteristics (e.g., severity, status, duration). We required a sample of > 200 participants to satisfy the COSMIN recommendation that the sample size for assessment of construct validity should be five times the number of items and > 100 [21]. We administered the AxEL-Q to consenting participants a second time to obtain retest data.

We recruited participants from:

- Databases of people with low back pain who had expressed an interest in research. We had access to a database of people with low back pain recruited from primary care centres in the Sydney metropolitan area. These people were not included in previous research studies with our group but consented to future contact regarding research studies.
- Social media. We posted an invitation to participate in this research study on social media platforms, Facebook, and Twitter.

### Procedure

Participants accessed the questionnaire via email or by following a link on social media. The questionnaire was in electronic form and was produced following our institutions’ data security standards using Qualtrics [30]. Participants had access to an informed consent document and consented to be involved by proceeding with the survey. Participants were sent reminders to complete partially completed surveys on three occasions, if possible.

#### Measures

As well as the draft questionnaire participants completed demographic questions, including questions on age and sex and questions about the duration of low back pain and its intensity. In addition, participants completed two validated back pain questionnaires, the Back Beliefs Questionnaire (BBQ) [31] and the modified Pain Self-Efficacy Questionnaire (PSEQ) [32], to evaluate convergent validity. The BBQ is an instrument to measure beliefs and attitudes related to back pain [31]. The BBQ has 14 items, including five distractors (questions 4, 5, 7, 9 and 11) not included in the final score [31, 33]. Responses are scored on a 5-point Likert scale from completely disagree (1 point) to completely agree (5 points); the total score ranges from 9 to 45. Higher scores indicate more pessimistic beliefs about the consequences of low back pain. The modified PSEQ is an instrument based on the psychological construct of self-efficacy which refers to an individual’s belief in their capacity to execute a behaviour necessary to achieve a specific task [34]. The modified PSEQ measures how confident participants feel about completing certain activities despite their pain. The modified PSEQ used had three questions [32]. Responses are scored on a 7-point scale from not at all confident (0 points) to completely confident (6 points). The total score ranges from 0 to 18. Higher scores indicate greater self-efficacy despite low back pain. These questionnaires are available in Additional file 3.

To obtain test–retest data, we emailed participants the AxEL-Q 3 months after completing the questionnaire for the first time. We chose a 3 month time interval between test and retest to ensure the elapsed time period was long enough to prevent the recall of previous answers but short enough to assume that the participants’ attitude may not change. Similarly, although some participants’ attitudes may change depending on symptom trajectories, we expected attitude changes to correspond with changes on the modified PSEQ and used stable modified PSEQ scores as a reference. We recognise that 3-months was longer than commonly used intervals so to minimise variability we based stability on a small standard deviation. We considered a change on the modified PSEQ of less than 0.5 standard deviations over 3 months to be a stable score. We used the AxEL-Q responses from participants with stable PSEQ scores only to evaluate test–retest reliability and measurement error.

### Data analysis

We used descriptive statistics to characterise the sample. We reported means and standard deviations for continuous variables. We used frequencies and percentages to report categorical variables. We used the R environment for statistical computing to conduct the analyses [35]. We used *psych* package version 2.1.3 to conduct the factor analysis, *irr* package, version 0.84.1 to determine interclass correlation coefficient and *rel* package, version 1.4.2 to determine the standard error of measurement.

#### Construct validity

##### Structural validity

Structural validity involves evaluating how well the scoring structure of the instrument corresponds to the construct being measured [22]. We used exploratory factor analysis (EFA) to evaluate the structural validity and to reduce the number of items in the questionnaire draft. We explored scree plots, parallel analysis, and Kaiser criterion to determine the most appropriate number of factors in keeping with standard EFA procedures. We used maximum likelihood estimation with oblimin rotation for factor extraction [36]. We used factor loading patterns to identify and extract items. Correlations of items to factors of less than 0.5 were sequentially deleted [37]. We continued a data-driven iterative process to achieve simple structure. We measured fit adequacy using the Tucker Lewis index (TLI) and comparative fit index (CFI). The TFL is an incremental fit index whereby bigger values indicate better fit. Values larger than 0.95 are interpreted as acceptable fit [38]. Values for CFI range between 0.0 and 1.0, with values closer to 1.0 indicating good fit. A CFI value of ≥ 0.90 is recommended [39]. Root mean square error of approximation (RMSEA) is an index of the difference between the observed covariance matrix and the hypothesised covariance matrix. RMSEA values smaller than 0.06 indicate a good fit, between 0.06 and 0.10 a mediocre fit and above 0.10 a poor fit [39]. Similarly for the root mean square of the residual (RMSR) index, smaller values indicate a better fit [40].

##### Hypotheses testing for convergent validity

Convergent validity measures the degree to which the scores on the AxEL-Q are consistent with hypotheses based on the assumption that the questionnaire validly measures attitude toward first-line care of low back pain [22]. There is no ‘gold standard’ for measuring attitude toward first-line care, therefore we evaluated convergent validity by testing hypotheses about the size and direction of the relationship between the AxEL-Q and two other validated back pain questionnaires, the BBQ and the modified PSEQ. The data was analysed as continuous data because there were six response options on the semantic differential scales, using Pearson’s r to test correlations between the AxEL-Q and two scales. We considered correlation coefficients < 0.30 as weak, coefficients between 0.30 and 0.49 as moderate, and coefficients ≥ 0.50 as strong [41].

Higher scores on BBQ indicate more pessimistic beliefs about the consequences of low back pain, we hypothesised that those with more pessimistic beliefs would be less likely to have a positive attitude toward statements of education and advice to represent first-line care. Therefore, we hypothesised that responses on the AxEL-Q would be inversely correlated to responses on the BBQ. Both questionnaires are designed to measure different but theoretically overlapping constructs, so we expected the strength of this relationship to be moderate corresponding to a Pearson’s r of − 0.30 to − 0.49. Conversely, as higher scores on the modified PSEQ indicate more confidence despite low back pain, we hypothesised that people with higher pain related self-efficacy would have a positive attitude toward statements of education and advice to represent first-line care. Therefore, we expected that responses on the AxEL-Q would be positively correlated to responses on the modified PSEQ. Both questionnaires also measure different but theoretically overlapping constructs, so we expected the strength of this relationship to be moderate, corresponding to a Pearson’s r of 0.30–0.49.

#### Reliability

##### Internal consistency

Internal consistency measures the degree to which items on the same scale are interrelated [20]. Following the EFA, we calculated Cronbach’s α for each factor. We considered values of Cronbach’s α < 0.70 as inadequate, values between 0.70 and 0.79 as adequate, values between 0.80 and 0.89 as good, and values ≥ 0.90 as excellent [42].

##### Test–retest reliability

Test–retest reliability refers to the proportion of the total variance in the measurements due to true differences [22]. We used a two-way random-effects model, interclass correlation coefficient (ICC) (2,1), as the participants came from a random sample. This model allows the questionnaire to be generalisable to different cohorts [20], such as patients with low back pain attending tertiary care. An ICC score of ≥ 0.70 was considered adequate [43].

##### Measurement error

The Standard Error of Measurement (SEM) accounts for the systematic and random error of a participants’ score that is not attributed to true changes in the construct to be measured [22]. We calculated the SEM by calculating the square root of the test–retest variance plus the residual variance to account for any systematic differences between the testing sessions [44]. This method is analogous to the two-way random-effects model we used to calculate test–retest reliability [20]. Larger scores indicate large variability, and smaller scores indicate minimal variability between testing sessions.

##### Minimal detectable change (MDC)

MDC is the threshold for determining true changes beyond measurement error and was calculated based on the SEM of the test–retest reliability [22]. The MDC was calculated as $SEM \times z \times \sqrt{2}$, [20, 36, 45] where SEM is the standard error of measurement and z = 1.96 (z score for estimating a 95% confidence interval). We used the square root of two, because there was a total of two measurements for test–retest reliability.

#### Floor and ceiling effects

Floor and ceiling effects are present if the questionnaire scores are either too low or too high so that detecting a deterioration or improvement would not be possible [22]. We defined floor and ceiling effects present if more than 15% of the participants reported the worst (minimum) or best (maximum) possible score [45].

#### Labelling

We reviewed the questionnaire in the context of evidence-based theory to ensure relevance to the target population and to label the factors.

## Results

### Descriptive statistics

The development process resulted in a questionnaire draft with 40-items. We evaluated the questionnaire draft in a sample of 345 participants with low back pain. One hundred and seventy-four participants who provided an email address were administered the survey twice, including the AxEL-Q, BBQ and modified PSEQ. Seventy-one participants of those one hundred and seventy-four provided stable retest data based on their modified PSEQ score. Table 1 outlines the demographic details of the participants. There were incomplete items for 32 participants, less than 10% of the overall sample. As we had exceeded our required sample size [46], data from these participants were omitted, leaving data from 313 participants for analysis. For those that responded to the follow-up questionnaire there were no missing data.

**Table 1:** Descriptive statistics

Characteristic	Total sample, n = 313	Retest cohort, n = 71
Age in years, mean (SD)	48.9 (16.7)	53.8 (14.2)
Female, n (%)	205 (65%)	45 (63%)
Cultural background, n (%)^a^	45 (14%)	NA
Health literacy, mean (SD)^b^	4.6 (0.7)	NA
Number of sessions of moderate-intensity physical activity per week, mean (SD)^c^	0.8 (0.8)	NA
Number of sessions of vigorous-intensity physical activity per week, mean (SD)^d^	1.6 (1.0)	NA
Low back pain intensity, mean (SD)^e^	3.4 (1.0)	5.5 (2.3)
Chronic low back pain, n (%)	252 (81%)	45 (63%)
Back Beliefs, mean (SD)^f^	26.7 (7.1)	27.6 (7.5)
Pain Self Efficacy, mean (SD)^g^	12.7 (4.8)	12.5 (4.4)

*SD* standard deviation, *NA* not asked

^a^Measured by asking whether participants spoke a language other than English at home, with answer options either yes or no

^b^Measured by asking how confident you are filling out medical forms by yourself with a range from 0 (not at all confident) to 5 (extremely confident)

^c^Moderate-intensity physical activity described as; increases your heart rate or makes you breathe harder than normal. (e.g., carrying light loads, bicycling at a regular pace or doubles tennis)

^d^Vigorous-intensity physical activity described as; makes you sweat or puff and pant. (e.g., heavy lifting, digging, jogging, aerobics, or fast bicycling)

^e^Numeric rating scale with a range from 0 (no pain) to 10 (worst pain possible)

^f^Back Beliefs Questionnaire with a range from 9 (less pessimistic beliefs) to 45 (more pessimistic beliefs)

^g^Modified pain self-efficacy questionnaire with a range from 0 (low self-efficacy) to 18 (high self-efficacy)

### Construct validity

#### Structural validity

Additional file 4 outlines the exploratory factor analysis results, including the four different methods used to determine how many factors best describe the data. A 3-Factor model achieved the best balance of goodness of fit, validity, reliability, and fitted with evidence-based theory. A simple structure was achieved after five rounds. The three factors together explained 65% of the variance in the model. Factor 1 explained 30% of variance, Factor 2 18%, Factor 3 17%. The resulting questionnaire included 17 items, the score range for Factor 1 was 0–54, 0 being the minimum possible score and 54 the maximum possible score, for Factor 2 0–24 and Factor 3 0–24. Full details of the EFA results including fit statistics are available in Additional file 5.

#### Hypotheses testing for convergent validity

All factors were strongly inversely correlated with the BBQ, suggesting those with a negative attitude toward first-line care had more pessimistic beliefs about low back pain. All factors on the 3-Factor AxEL-Q were moderate to strongly positively correlated with the modified PSEQ, suggesting that those with a positive attitude toward first-line care had greater pain-related self-efficacy. These results are presented in Table 2.

**Table 2:** Results of reliability, measurement error, construct validity and ceiling and floor effect analyses for the AxEL-Q

3 Factor model	Construct validity		ICC^a^	SEM^b^	MDC^c^	Ceiling effects	Floor effects
	Back beliefs questionnaire	Modified pain self-efficacy questionnaire				% Scoring maximum score (%)	% Scoring minimum score
Factor 1	− 0.56^d^	0.55^d^	0.71^d^	5.59^d^	15.49	6.4	0
	(− 0.63 to − 0.48)	(0.46 to 0.62)					
	(0.58 to 0.81)	(4.63 to 6.54)					
Factor 2	− 0.50^d^	0.37^d^	0.73^d^	3.51^d^	9.73	2.9	5.1%
	(− 0.58 to − 0.41)	(0.27 to 0.46)					
	(0.60 to 0.82)	(2.96 to 4.06)					
Factor 3	− 0.50^d^	0.35^d^	0.79^d^	2.68^d^	7.43	4.8	1.3%
	(− 0.58 to − 0.41)	(0.24 to 0.44)					
	(0.64 to 0.87)	(2.23 to 3.12)					

All effects are presented with their 95% confidence intervals

^a^Interclass correlation coefficient

^b^Standard error of measurement

^c^Minimal detectable change

^d^*p* < 0.01

### Reliability

#### Internal consistency

Each factor demonstrated good to excellent values of Cronbach’s α; Factor 1 α = 0.92, Factor 2 α = 0.91 and Factor 3 α = 0.90.

#### ICC, SEM and MDC

The 3-Factor model demonstrated adequate ICC, but moderate to high values of SEM and MDC. Table 2 outlines the ICC, SEM and MDC.

### Floor and ceiling effects

The 3-Factor model did not display a floor or ceiling effect. Table 2 outlines the percentage of participants who scored the maximum or minimum score for each factor.

### Labelling

**Fig. 2:**
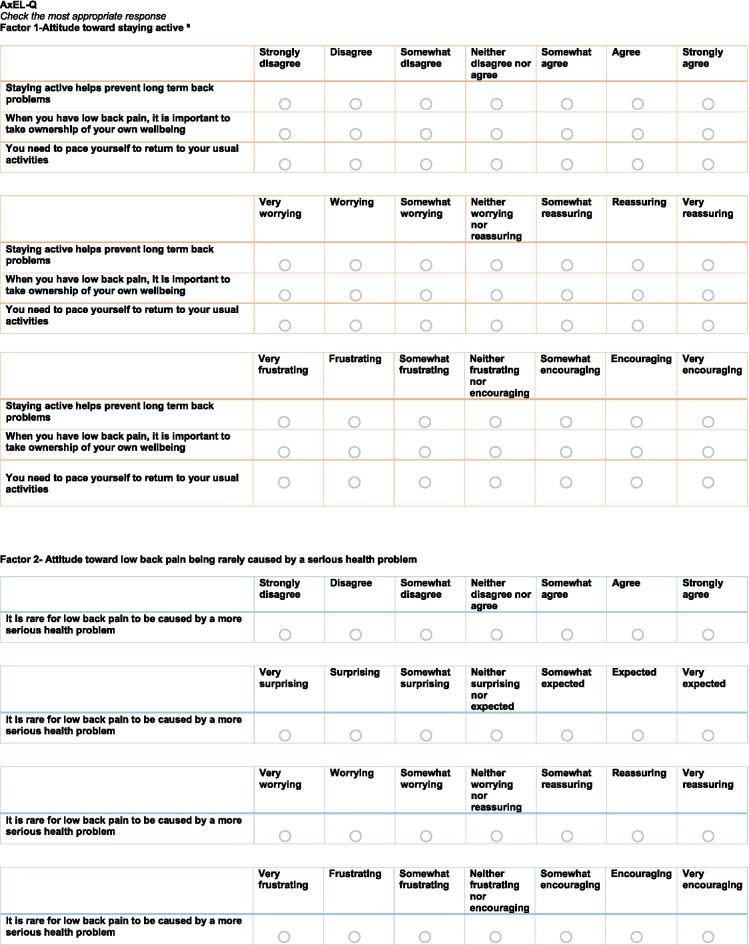

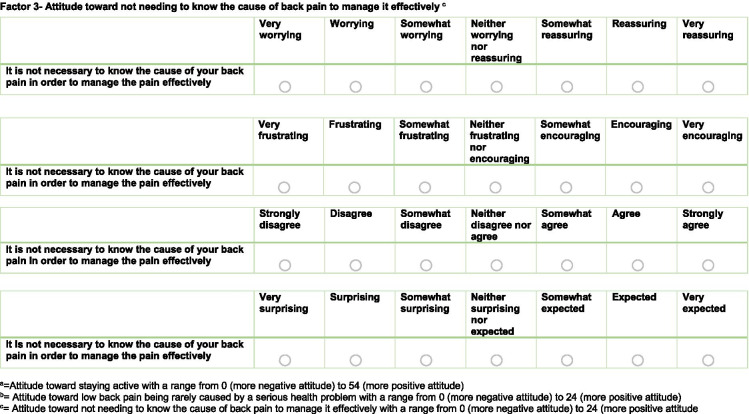
AxEL-Q

The three factors complemented clinical practice guidelines for the management of low back pain and were named accordingly Factor 1; *Attitude toward staying active*, Factor 2; *Attitude toward low back pain being rarely caused by a serious health problem,* Factor 3; *Attitude toward not needing to know the cause of back pain to manage it effectively*. The full details of the final-version of the 3-Factor model (titled the AxEL-Q) are available in Fig. 2.

## Discussion

The AxEL-Q provides clinicians with a valid and reliable tool to understand patient attitudes toward first-line care for low back pain. Each subscale demonstrated acceptable construct validity and reliability, meaning that the questionnaire could be used as a whole or as individual subscales. The independent utility of the subscales allows clinicians to understand attitudes toward distinct aspects of first line care in people with low back pain.

The AxEL-Q may help inform and guide the management of people presenting with low back pain. This questionnaire can provide clinicians with an insight to effectively implement current recommendations at the outset of a clinical encounter. For example, if a patient scores high on ‘the attitude towards not needing to know the cause of back pain to manage it effectively, the clinician could devote more time during the consultation towards other aspects of first-line care such as promoting physical activity. Whereas if a patient has a negative attitude towards this factor, the clinician could prioritise time and resources to discuss how imaging may do more harm than good when serious conditions are not suspected and is likely to prolong recovery [47]. Future research would be needed to evaluate the effectiveness of using the AxEL-Q in this way.

Five statements that represented first line care were omitted as part of the EFA process but may still have some relevance for clinical practice. We conducted a separate analysis to understand if any of the 10 statements that were included in the questionnaire draft were predictive of self-managing low back pain in people with low back pain of different durations. Our findings indicate that people’s attitude toward certain key messages was predictive of their intention to self-manage, but which message and the size of the effect was dependent on whether someone had low back pain and for how long [48].

### Content validity

We comprehensively represented first-line care for low back pain by developing the questionnaire items relative to ten expert and consumer endorsed advice and education statements. Although the questionnaire was developed within the COSMIN framework, we did not conduct qualitative testing before testing construct validity. Instead, we assumed relevance, comprehensiveness, and comprehensibility, but qualitative testing would have confirmed these assumptions. We acknowledge that this is a limitation of this study.

### Construct validity

The exploratory factor analysis demonstrated a 3-Factor model in keeping with the conceptual framework that attitude toward first-line care for low back pain is a multi-dimensional construct, measured indirectly using multiple observable items. Five statements that represented first line care were omitted as part of the EFA process but may still have some relevance for clinical practice.

The AxEL-Q demonstrated good construct validity suggesting that it measures what it is designed to measure, in keeping with prior hypotheses. The strength of the relationship with the BBQ and modified PSEQ supports that unhelpful beliefs underpin unhelpful attitudes, and through the relationship with self-efficacy it suggests the AxEL-Q has promise at predicting future behaviour. In general these relationships suggest the value of the AxEL-Q to complement questionnaires commonly used with people with low back pain by evaluating attitudes toward specific elements of first-care that would not be captured in general questionnaires.

### Reliability

Each factor on the AxEL-Q demonstrated good internal consistency. The ICC is adequate, meaning that it can differentiate between people while remaining relatively stable between testing sessions. The SEM ranges from 10.4% of the overall score for Factor 1–14.6% for Factor 2; these values are in keeping with other validated patient-reported outcomes [36]. The MDC was high; for example, a change score of more than 15 points for Factor 1 (> 28% change) would be necessary to determine a true change. A high MDC is a common issue with patient-reported questionnaires [36].

### Floor and ceiling effects

6.4% of responses recorded the maximum possible score. The included participants expressed an interest in health research. These participants may be more likely to have a positive attitude toward first-line care as they had volunteered for research that included an education component. Floor and ceiling effects are sensitive to the population under study so the result might be different in a clinical population.

### Strengths and weaknesses

A key strength of this study is the rigorous method used to develop the AxEL-Q and evaluate its clinimetric performance, in line with the COSMIN recommendations [20, 22, 23]. Other validated questionnaires designed to measure attitude measure levels of agreement with particular statements [15, 17, 18, 49], in comparison the AxEL-Q appears to be a more sophisticated measure that considers both cognitive and affective components of attitude. However, with up to seventeen questions, we do not anticipate excessive participant burden.

This study has some weaknesses. Attitude is only one component of intention, and therefore provides a limited insight to the subsequent behaviour. The theory of planned behaviour model suggests that perceived behavioural control and subjective norms together with attitude shape an individuals’ behavioural intentions [50]. Evaluating the clinical utility to understand if attitudes actually correspond with the predicted behaviour is necessary. Second, qualitative testing is an important aspect of designing questionnaires. We undertook a comprehensive process to design the questionnaire but specific qualitative testing would have strengthened the results. Third, there was a 3-month interval between the first and retest data collection period. We allowed this interval because attitude and self-efficacy are considered stable constructs and we anticipated that this time frame was necessary to prevent recall of initial responses. However we acknowledge that intervals from 14 days to 1 month are more commonly used. Fourth, we had incomplete data for 32 participants. We suggest this was due to questionnaire fatigue due to the large number of questions. Fifth, the implication of the SEM and the MCD will not be evident until the AxEL-Q is used to assess a change in attitude as part of an intervention or longitudinal study. These study designs where we anticipate a change over time or between groups will provide an understanding of how realistic it is to determine a true change, i.e. a change that is greater than the value of the SEM. Future studies are needed to evaluate the measurement properties of the AxEL Questionnaire by strictly focusing on Clinimetric Patient-Reported Outcome Measures (CLIPROM) criteria that represent a step forward to the development of new clinimetric indices and validation process of existing rating scales to be used in clinical research and practice [51].

## Conclusion

The underlying theoretical basis of the AxEL-Q is that attitude toward first-line care predicts intention to behave in line with that recommendation. Further evaluation should test the responsiveness of the AxEL-Q in a longitudinal design such as in a randomised controlled trial and calculating the minimally clinically important change could improve the interpretability of the AxEL-Q.

Despite these limitations the questionnaire enables clinicians to efficiently personalise first-line care to ensure that it aligns with the patient’s attitudes which may improve patient satisfaction, and potentially improve treatment outcomes.

## Supplementary Information

**Additional file 1**. Development of the AxEL-Q.

**Additional file 2**. Questionnaire draft.

**Additional file 3**. Back Beliefs Questionnaire & the Modified Pain Self-Efficacy Questionnaire.

**Additional file 4**. Exploratory Factor Analysis (EFA) results.

**Additional file 5**. The three-factor Model.

## Data Availability

The datasets used and analysed during the current study are available from the corresponding author on reasonable request. The study protocol including the analysis plan is publicly available on the Open Science Framework https://osf.io/9wncz/ Data will be made publicly available on the Open Science Framework on publication.
