# Supplementary material for: Development and measurement properties of the AxEL (attitude toward education and advice for low-back-pain) questionnaire
Source: Health Qual Life Outcomes. 2022 Jan 10;20:4. doi: 10.1186/s12955-021-01908-4 (PMC8744221; doi:10.1186/s12955-021-01908-4)
Supplement: Supplementary file 1 — Additional file 1. Development of the AxEL-Q. [file 12955_2021_1908_MOESM1_ESM.docx]

# Supplementary Material 1- Creating semantic scales.

## Step 1- List all adjectives that appear at least twice in response to open-ended questions.

Reassuring

False

True

Helpful/unhelpful

Don’t believe/unbelievable

Incorrect

Surprising

Helpful

Informative

Agree/disagree

Motivated/ demotivating

Encouraging

Understand/don’t understand

True

Reassuring

Confusing

Frustrated

Happy/ happiness

Useful

Annoying

Frustrated

Surprising

Clear

Helpful

Useful

Annoying

Interesting

Sounds good

Reasonable

Erroneous

Accurate

doubtful

Hopeful

Positive

Dismissive

Anxious

Relieved

Not confident

## Step 2- Identify antonyms

| Reassuring  Relieved | Worrying  Worried |
| --- | --- |
| True  Agree  Accurate | False  Disagree  Incorrect  Erroneous |
| Helpful  Useful | Unhelpful |
| Surprising  Doubtful | Expected  Certain |
| Informative  Interesting | Confusing  Boring |
| Motivating | Discouraging |
| Encouraging | Discouraging |
| Understandable  Clear | Irrational  Confusing |
| Frustrated  Annoying | Encouraged  Helpful |
| Reasonable | Unreasonable |
| Hopeful | Depressed |
| Positive | Negative |
| Confident | Not-confident  Anxious |
| Dismissive | Well cared for |
| Happy | Sad |
| Believe | Disbelieve |

## Step 3- Create semantic scale anchors

Believe----Disbelieve

Reassured---- Worried

Motivating-----Discouraging

Expected-----Surprising

Helpful-----Unhelpful

Agree-------Disagree

Informative-----Confusing

Interesting----Boring

Understandable-----Irrational

Encouraging----Frustrated

Hopeful-----Depressed

Confident-----Anxious

Well cared for-----Dismissed

Happy------Sad

## Step 4- Correlation matrix of adjectives pairs for each of the 10 statements to identify highly correlated pairs

Correlations coefficients for adjective pairs toward; it is not necessary to know the specific cause of your back pain in order to manage the pain effectively

|  | Worried-Reassured | Discouraged-Motivated | Surprised-Expected | Unhelpful-helpful | Disagree-agree | Confused-informed | Boring-Interesting | Irrational-Understandable | Frustrated-encouraged | Depressed-hopeful | Anxious-confident | Sad-Happy | Not cared for- Well cared for |
| --- | --- | --- | --- | --- | --- | --- | --- | --- | --- | --- | --- | --- | --- |
| Worried-reassured | 1 | .826** | .708** | .810** | .702** | .815** | .780** | .666** | .829** | .793** | .764** | .793** | .735** |
| Discouraged-motivated |  | 1 | .533* | .817** | .705** | .853** | .726** | .831** | .737** | .730** | .740** | .767** | .712** |
| Surprised-expected |  |  | 1 | .347 | .624** | .643** | .450 | .613** | .594* | .423 | .481 | .531* | .500* |
| Unhelpful-helpful |  |  |  | 1 | .617** | .821** | .828** | .677** | .799** | .800** | .701** | .745** | .707** |
| Disagree-agree |  |  |  |  | 1 | .912** | .714** | .845** | .662** | .822** | .829** | .845** | .732** |
| Confused-informed |  |  |  |  |  | 1 | .785** | .835** | .816** | .919** | .904** | .941** | .859** |
| Boring-interesting |  |  |  |  |  |  | 1 | .749** | .849** | .847** | .807** | .790** | .647** |
| Irrational-understandable |  |  |  |  |  |  |  | 1 | .644** | .707** | .762** | .763** | .589* |
| Frustrated-encouraged |  |  |  |  |  |  |  |  | 1 | .843** | 794** | .769** | .791** |
| Depressed-hopeful |  |  |  |  |  |  |  |  |  | 1 | .961** | .953** | .881** |
| Anxious-confident |  |  |  |  |  |  |  |  |  |  | 1 | .977* | .889** |
| Sad-happy |  |  |  |  |  |  |  |  |  |  |  | 1 | .897** |
| Not cared for-cared for |  |  |  |  |  |  |  |  |  |  |  |  | 1 |

** p<0.01 *p<.0.05

Correlations coefficients for adjective pairs toward; it is rare for low back pain to be caused by a more serious health

|  | Worried-Reassured | Discouraged-Motivated | Surprised-Expected | Unhelpful-helpful | Disagree-agree | Confused-informed | Boring-Interesting | Irrational-Understandable | Frustrated-encouraged | Depressed-hopeful | Anxious-confident | Sad-Happy | Not cared for- Well cared for |
| --- | --- | --- | --- | --- | --- | --- | --- | --- | --- | --- | --- | --- | --- |
| Worried-reassured | 1 | .833^**^ | .521^*^ | .666^**^ | .150 | .408 | .565^*^ | .582^*^ | .491^*^ | .175 | .006 | .275 | .391 |
| Discouraged-motivated |  | 1 | .439 | .815^**^ | .070 | .411 | .705^**^ | .363 | .484^*^ | .283 | .137 | .470 | .537* |
| Surprised-expected |  |  | 1 | .394 | .535^*^ | .525^*^ | -.045 | .707^**^ | .448 | .086 | -.046 | .139 | -.050 |
| Unhelpful-helpful |  |  |  | 1 | -.045 | .604^*^ | .630^**^ | .449 | .677^**^ | .592^*^ | .400 | .589^*^ | .101 |
| Disagree-agree |  |  |  |  | 1 | .384 | -.107 | .415 | .072 | .096 | .185 | .112 | .641** |
| Confused-informed |  |  |  |  |  | 1 | .349 | .727^**^ | .634^**^ | .622^**^ | .401 | .367 | .244 |
| Boring-interesting |  |  |  |  |  |  | 1 | .136 | .442 | .436 | .230 | .445 | .614** |
| Irrational-understandable |  |  |  |  |  |  |  | 1 | .591^*^ | .422 | .161 | .079 | .634** |
| Frustrated-encouraged |  |  |  |  |  |  |  |  | 1 | .686^**^ | .691^**^ | .461 | .364 |
| Depressed-hopeful |  |  |  |  |  |  |  |  |  | 1 | .727^**^ | .356 | .675** |
| Anxious-confident |  |  |  |  |  |  |  |  |  |  | 1 | .557^*^ | .722** |
| Sad-happy |  |  |  |  |  |  |  |  |  |  |  | 1 | .735** |
| Not cared for-cared for |  |  |  |  |  |  |  |  |  |  |  |  | 1 |

** p<0.01 *p<.0.05

Correlations coefficients for adjective pairs toward; you need to pace yourself to return to your usual activities

|  | Worried-Reassured | Discouraged-Motivated | Surprised-Expected | Unhelpful-helpful | Disagree-agree | Confused-informed | Boring-Interesting | Irrational-Understandable | Frustrated-encouraged | Depressed-hopeful | Anxious-confident | Sad-Happy | Not cared for- Well cared for |
| --- | --- | --- | --- | --- | --- | --- | --- | --- | --- | --- | --- | --- | --- |
| Worried-reassured | 1 | .826** | .708** | .810** | .702** | .815** | .780** | .666** | .829** | .793** | .764** | .793** | .735** |
| Discouraged-motivated |  | 1 | .533* | .817** | .705** | .853** | .726** | .831** | .737** | .730** | .740** | .767** | .712** |
| Surprised-expected |  |  | 1 | .347 | .624** | .643** | .450 | .613** | .594* | .423 | .481 | .531* | .500* |
| Unhelpful-helpful |  |  |  | 1 | .617** | .821** | .828** | .677** | .799** | .800** | .701** | .745** | .707** |
| Disagree-agree |  |  |  |  | 1 | .912** | .714** | .845** | .662** | .822** | .829** | .845** | .732** |
| Confused-informed |  |  |  |  |  | 1 | .785** | .835** | .816** | .919** | .904** | .941** | .859** |
| Boring-interesting |  |  |  |  |  |  | 1 | .749** | .849** | .847** | .807** | .790** | .647** |
| Irrational-understandable |  |  |  |  |  |  |  | 1 | .644** | .707** | .762** | .763** | .589* |
| Frustrated-encouraged |  |  |  |  |  |  |  |  | 1 | .843** | 794** | .769** | .791** |
| Depressed-hopeful |  |  |  |  |  |  |  |  |  | 1 | .961** | .953** | .881** |
| Anxious-confident |  |  |  |  |  |  |  |  |  |  | 1 | .977* | .889** |
| Sad-happy |  |  |  |  |  |  |  |  |  |  |  | 1 | .897** |
| Not cared for-cared for |  |  |  |  |  |  |  |  |  |  |  |  | 1 |

** p<0.01 *p<.0.05

Correlations coefficients for adjective pairs toward; when you have low back pain it is important to take ownership of your own well being

|  | Worried-Reassured | Discouraged-Motivated | Surprised-Expected | Unhelpful-helpful | Disagree-agree | Confused-informed | Boring-Interesting | Irrational-Understandable | Frustrated-encouraged | Depressed-hopeful | Anxious-confident | Sad-Happy | Not cared for- Well cared for |
| --- | --- | --- | --- | --- | --- | --- | --- | --- | --- | --- | --- | --- | --- |
| Worried-reassured | 1 | .788^**^ | .391 | .929^**^ | .616^**^ | .753^**^ | .759^**^ | .777^**^ | .550^*^ | .558^*^ | .738^**^ | .634^**^ | .449 |
| Discouraged-motivated |  | 1 | .481^*^ | .787^**^ | .782^**^ | .840^**^ | .926^**^ | .837^**^ | .707^**^ | .717^**^ | .720^**^ | .710^**^ | .575* |
| Surprised-expected |  |  | 1 | .255 | .596^**^ | .497^*^ | .481 | .591^*^ | .409 | .456 | .453 | .514^*^ | .447 |
| Unhelpful-helpful |  |  |  | 1 | .571^*^ | .802^**^ | .824^**^ | .814^**^ | .609^**^ | .675^**^ | .747^**^ | .674^**^ | .490* |
| Disagree-agree |  |  |  |  | 1 | .739^**^ | .859^**^ | .910^**^ | .737^**^ | .690^**^ | .697^**^ | .806^**^ | .719* |
| Confused-informed |  |  |  |  |  | 1 | .871^**^ | .909^**^ | .818^**^ | .913^**^ | .852^**^ | .784^**^ | .794** |
| Boring-interesting |  |  |  |  |  |  | 1 | .854^**^ | .840^**^ | .751^**^ | .758^**^ | .774^**^ | .749** |
| Irrational-understandable |  |  |  |  |  |  |  | 1 | .814^**^ | .855^**^ | .841^**^ | .852^**^ | .761** |
| Frustrated-encouraged |  |  |  |  |  |  |  |  | 1 | .743^**^ | .776^**^ | .715^**^ | .879** |
| Depressed-hopeful |  |  |  |  |  |  |  |  |  | 1 | .819^**^ | .831^**^ | .771** |
| Anxious-confident |  |  |  |  |  |  |  |  |  |  | 1 | .756^**^ | .749** |
| Sad-happy |  |  |  |  |  |  |  |  |  |  |  | 1 | .755** |
| Not cared for-cared for |  |  |  |  |  |  |  |  |  |  |  |  | 1 |

** p<0.01 *p<.0.05

Correlations coefficients for adjective pairs toward; don’t take back pain lying down

|  | Worried-Reassured | Discouraged-Motivated | Surprised-Expected | Unhelpful-helpful | Disagree-agree | Confused-informed | Boring-Interesting | Irrational-Understandable | Frustrated-encouraged | Depressed-hopeful | Anxious-confident | Sad-Happy | Not cared for- Well cared for |
| --- | --- | --- | --- | --- | --- | --- | --- | --- | --- | --- | --- | --- | --- |
| Worried-reassured | 1 | .651^**^ | .035 | .505^*^ | .609^**^ | .466 | .770^**^ | .519^*^ | .424 | .589^*^ | .435 | .686^**^ | .326 |
| Discouraged-motivated |  | 1 | .303 | .857^**^ | .802^**^ | .745^**^ | .813^**^ | .792^**^ | .801^**^ | .852^**^ | .771^**^ | .889^**^ | .606** |
| Surprised-expected |  |  | 1 | .192 | .382 | .335 | .073 | .181 | .410 | .299 | .321 | .211 | .416* |
| Unhelpful-helpful |  |  |  | 1 | .508^*^ | .735^**^ | .823^**^ | .663^**^ | .791^**^ | .815^**^ | .620^**^ | .813^**^ | .708** |
| Disagree-agree |  |  |  |  | 1 | .646^**^ | .675^**^ | .754^**^ | .683^**^ | .748^**^ | .795^**^ | .797^**^ | .428 |
| Confused-informed |  |  |  |  |  | 1 | .699^**^ | .886^**^ | .755^**^ | .897^**^ | .778^**^ | .774^**^ | .590* |
| Boring-interesting |  |  |  |  |  |  | 1 | .756^**^ | .708^**^ | .825^**^ | .730^**^ | .847^**^ | .682** |
| Irrational-understandable |  |  |  |  |  |  |  | 1 | .782^**^ | .907^**^ | .877^**^ | .904^**^ | .517* |
| Frustrated-encouraged |  |  |  |  |  |  |  |  | 1 | .852^**^ | .887^**^ | .785^**^ | .801** |
| Depressed-hopeful |  |  |  |  |  |  |  |  |  | 1 | .886^**^ | .895^**^ | .673** |
| Anxious-confident |  |  |  |  |  |  |  |  |  |  | 1 | .791^**^ | .675** |
| Sad-happy |  |  |  |  |  |  |  |  |  |  |  | 1 | .597* |
| Not cared for-cared for |  |  |  |  |  |  |  |  |  |  |  |  | 1 |

** p<0.01 *p<.0.05

Correlations coefficients for adjective pairs toward; your pain may not necessarily be related to the extent of damage in your back. Hurt does not necessarily mean harm

|  | Worried-Reassured | Discouraged-Motivated | Surprised-Expected | Unhelpful-helpful | Disagree-agree | Confused-informed | Boring-Interesting | Irrational-Understandable | Frustrated-encouraged | Depressed-hopeful | Anxious-confident | Sad-Happy | Not cared for- Well cared for |
| --- | --- | --- | --- | --- | --- | --- | --- | --- | --- | --- | --- | --- | --- |
| Worried-reassured | 1 | .891^**^ | .240 | .921^**^ | .610^**^ | .724^**^ | .857^**^ | .480 | .579^*^ | .723^**^ | .549^*^ | .552^*^ | .551* |
| Discouraged-motivated |  | 1 | .211 | .851^**^ | .486^*^ | .610^**^ | .847^**^ | .320 | .541^*^ | .639^**^ | .465 | .492^*^ | .516* |
| Surprised-expected |  |  | 1 | .145 | .457 | -.165 | .122 | .256 | -.061 | .090 | .189 | .008 | .137 |
| Unhelpful-helpful |  |  |  | 1 | .551^*^ | .652^**^ | .841^**^ | .377 | .585^*^ | .772^**^ | .565^*^ | .629^**^ | .578* |
| Disagree-agree |  |  |  |  | 1 | .554* | .630^**^ | .637^**^ | .397 | .482 | .346 | .336 | .621** |
| Confused-informed |  |  |  |  |  | 1 | .687^**^ | .614^**^ | .630^**^ | .528^*^ | .379 | .370 | .456 |
| Boring-interesting |  |  |  |  |  |  | 1 | .317 | .658^**^ | .711^**^ | .584^*^ | .739^**^ | .705** |
| Irrational-understandable |  |  |  |  |  |  |  | 1 | .561^*^ | .489^*^ | .385 | .125 | .358 |
| Frustrated-encouraged |  |  |  |  |  |  |  |  | 1 | .863^**^ | .784^**^ | .607^**^ | .778** |
| Depressed-hopeful |  |  |  |  |  |  |  |  |  | 1 | .808^**^ | .696^**^ | .766** |
| Anxious-confident |  |  |  |  |  |  |  |  |  |  | 1 | .747^**^ | .751** |
| Sad-happy |  |  |  |  |  |  |  |  |  |  |  | 1 | .672** |
| Not cared for-cared for |  |  |  |  |  |  |  |  |  |  |  |  | 1 |

** p<0.01 *p<.0.05

Correlations coefficients for adjective pairs toward; in around 95% of cases it is not possible to pinpoint the cause of back pain

|  | Worried-Reassured | Discouraged-Motivated | Surprised-Expected | Unhelpful-helpful | Disagree-agree | Confused-informed | Boring-Interesting | Irrational-Understandable | Frustrated-encouraged | Depressed-hopeful | Anxious-confident | Sad-Happy | Not cared for- Well cared for |
| --- | --- | --- | --- | --- | --- | --- | --- | --- | --- | --- | --- | --- | --- |
| Worried-reassured | 1 | .354 | -.098 | .393 | -.038 | -.083 | .477 | .008 | .399 | .460 | .563^*^ | .463 | .435 |
| Discouraged-motivated |  | 1 | .039 | .728^**^ | .311 | .585^*^ | .529^*^ | .446 | .709^**^ | .747^**^ | .606^**^ | .689^**^ | .586** |
| Surprised-expected |  |  | 1 | .140 | .394 | -.326 | -.269 | -.244 | -.028 | -.147 | -.357 | -.434 | -.233 |
| Unhelpful-helpful |  |  |  | 1 | .433 | .436 | .681^**^ | .158 | .352 | .557^*^ | .341 | .427 | .444 |
| Disagree-agree |  |  |  |  | 1 | .304 | .430 | .072 | .081 | .009 | .030 | .030 | .300 |
| Confused-informed |  |  |  |  |  | 1 | .478 | .713^**^ | .271 | .427 | .418 | .415 | .366 |
| Boring-interesting |  |  |  |  |  |  | 1 | .270 | .386 | .487^*^ | .481 | .542^*^ | .524* |
| Irrational-understandable |  |  |  |  |  |  |  | 1 | .437 | .612^**^ | .541^*^ | .519^*^ | .546* |
| Frustrated-encouraged |  |  |  |  |  |  |  |  | 1 | .577^*^ | .630^**^ | .629^**^ | .649** |
| Depressed-hopeful |  |  |  |  |  |  |  |  |  | 1 | .581^*^ | .780^**^ | .700** |
| Anxious-confident |  |  |  |  |  |  |  |  |  |  | 1 | .825^**^ | .695** |
| Sad-happy |  |  |  |  |  |  |  |  |  |  |  | 1 | .797** |
| Not cared for-cared for |  |  |  |  |  |  |  |  |  |  |  |  | 1 |

** p<0.01 *p<.0.05

Correlations coefficients for adjective pairs toward; most people find their low back pain settles down over a short period of time. If your back pain persists and is worrying you, consult a health professional.

|  | Worried-Reassured | Discouraged-Motivated | Surprised-Expected | Unhelpful-helpful | Disagree-agree | Confused-informed | Boring-Interesting | Irrational-Understandable | Frustrated-encouraged | Depressed-hopeful | Anxious-confident | Sad-Happy | Not cared for- Well cared for |
| --- | --- | --- | --- | --- | --- | --- | --- | --- | --- | --- | --- | --- | --- |
| Worried-reassured | 1 | .700^**^ | .306 | .792^**^ | .213 | .572^*^ | .676^**^ | .350 | .705^**^ | .436 | .543^*^ | .602^*^ | .626** |
| Discouraged-motivated |  | 1 | .345 | .559^*^ | .155 | .088 | .325 | -.160 | .239 | .098 | .257 | .283 | .219 |
| Surprised-expected |  |  | 1 | .216 | .413 | -.151 | -.254 | .050 | -.040 | -.266 | .003 | -.060 | -.221 |
| Unhelpful-helpful |  |  |  | 1 | .344 | .404 | .592^*^ | .123 | .433 | .409 | .403 | .601^*^ | .460 |
| Disagree-agree |  |  |  |  | 1 | .368 | .234 | .388 | .418 | .396 | .563^*^ | .578^*^ | .247 |
| Confused-informed |  |  |  |  |  | 1 | .764^**^ | .807^**^ | .723^**^ | .664^**^ | .742^**^ | .723^**^ | .762** |
| Boring-interesting |  |  |  |  |  |  | 1 | .424 | .704^**^ | .696^**^ | .596^*^ | .697^**^ | .809** |
| Irrational-understandable |  |  |  |  |  |  |  | 1 | .615^**^ | .615^**^ | .651^**^ | .575^*^ | .597* |
| Frustrated-encouraged |  |  |  |  |  |  |  |  | 1 | .709^**^ | .789^**^ | .775^**^ | .849** |
| Depressed-hopeful |  |  |  |  |  |  |  |  |  | 1 | .704^**^ | .840^**^ | .679** |
| Anxious-confident |  |  |  |  |  |  |  |  |  |  | 1 | .911^**^ | .775* |
| Sad-happy |  |  |  |  |  |  |  |  |  |  |  | 1 | .731** |
| Not cared for-cared for |  |  |  |  |  |  |  |  |  |  |  |  | 1 |

** p<0.01 *p<.0.05

Correlations coefficients for adjective pairs toward; when you have back pain staying active is important. You need to pace yourself to return to your usual activities.

|  | Worried-Reassured | Discouraged-Motivated | Surprised-Expected | Unhelpful-helpful | Disagree-agree | Confused-informed | Boring-Interesting | Irrational-Understandable | Frustrated-encouraged | Depressed-hopeful | Anxious-confident | Sad-Happy | Not cared for- Well cared for |
| --- | --- | --- | --- | --- | --- | --- | --- | --- | --- | --- | --- | --- | --- |
| Worried-reassured | 1 | .824^**^ | .238 | .907^**^ | .609^**^ | .774^**^ | .868^**^ | .832^**^ | .686^**^ | .743^**^ | .623^**^ | .748^**^ | .819** |
| Discouraged-motivated |  | 1 | .401 | .886^**^ | .560^*^ | .813^**^ | .855^**^ | .871^**^ | .721^**^ | .786^**^ | .742^**^ | .756^**^ | .840** |
| Surprised-expected |  |  | 1 | .182 | .429 | .534^*^ | .361 | .492^*^ | .368 | .361 | .367 | .302 | .361 |
| Unhelpful-helpful |  |  |  | 1 | .589^*^ | .753^**^ | .922^**^ | .849^**^ | .723^**^ | .810^**^ | .714^**^ | .743^**^ | .809** |
| Disagree-agree |  |  |  |  | 1 | .628^**^ | .776^**^ | .711^**^ | .790^**^ | .862^**^ | .831^**^ | .871^**^ | .832** |
| Confused-informed |  |  |  |  |  | 1 | .808^**^ | .938^**^ | .655^**^ | .804^**^ | .721^**^ | .739^**^ | .835** |
| Boring-interesting |  |  |  |  |  |  | 1 | .912^**^ | .794^**^ | .872^**^ | .763^**^ | .832^**^ | .915** |
| Irrational-understandable |  |  |  |  |  |  |  | 1 | .658^**^ | .808^**^ | .747^**^ | .778^**^ | .867** |
| Frustrated-encouraged |  |  |  |  |  |  |  |  | 1 | .890^**^ | .806^**^ | .806^**^ | .825** |
| Depressed-hopeful |  |  |  |  |  |  |  |  |  | 1 | .923^**^ | .884^**^ | .924** |
| Anxious-confident |  |  |  |  |  |  |  |  |  |  | 1 | .889^**^ | .834** |
| Sad-happy |  |  |  |  |  |  |  |  |  |  |  | 1 | .938** |
| Not cared for-cared for |  |  |  |  |  |  |  |  |  |  |  |  | 1 |

** p<0.01 *p<.0.05

Correlations coefficients for adjective pairs toward; Imaging is usually not need in the majority of cases of low back pain, particularly when your pain has been present for less than 6 weeks. Talk to your doctor about this.

|  | Worried-Reassured | Discouraged-Motivated | Surprised-Expected | Unhelpful-helpful | Disagree-agree | Confused-informed | Boring-Interesting | Irrational-Understandable | Frustrated-encouraged | Depressed-hopeful | Anxious-confident | Sad-Happy | Not cared for- Well cared for |
| --- | --- | --- | --- | --- | --- | --- | --- | --- | --- | --- | --- | --- | --- |
| Worried-reassured | 1 | .392 | .093 | .508^*^ | .494^*^ | .692^**^ | .687^**^ | .546^*^ | .387 | .630^**^ | .607^**^ | .680^**^ | .842** |
| Discouraged-motivated |  | 1 | .272 | .310 | .362 | .683^**^ | .180 | .399 | .083 | .541^*^ | .259 | .500^*^ | .532* |
| Surprised-expected |  |  | 1 | .449 | .471^*^ | .470 | .046 | .469 | .142 | .413 | .480 | .460 | .306 |
| Unhelpful-helpful |  |  |  | 1 | .718^**^ | .715^**^ | .851^**^ | .617^**^ | .526^*^ | .543^*^ | .862^**^ | .820^**^ | .826** |
| Disagree-agree |  |  |  |  | 1 | .719^**^ | .497^*^ | .763^**^ | .381 | .406 | .777^**^ | .509^*^ | .755** |
| Confused-informed |  |  |  |  |  | 1 | .577^*^ | .754^**^ | .388 | .451 | .616^**^ | .655^**^ | .831** |
| Boring-interesting |  |  |  |  |  |  | 1 | .427 | .373 | .421 | .663^**^ | .738^**^ | .744** |
| Irrational-understandable |  |  |  |  |  |  |  | 1 | .498^*^ | .360 | .564^*^ | .420 | .695** |
| Frustrated-encouraged |  |  |  |  |  |  |  |  | 1 | -.040 | .287 | .143 | .356 |
| Depressed-hopeful |  |  |  |  |  |  |  |  |  | 1 | .682^**^ | .841^**^ | .665** |
| Anxious-confident |  |  |  |  |  |  |  |  |  |  | 1 | .793^**^ | .783** |
| Sad-happy |  |  |  |  |  |  |  |  |  |  |  | 1 | .799** |
| Not cared for-cared for |  |  |  |  |  |  |  |  |  |  |  |  | 1 |

** p<0.01 *p<.0.05

## Step 5- Remove highly correlated pairs

Discouraged Motivated

Unhelpful helpful

Confusing Informative

Boring interesting

Irrational Understandable

Depressing Hopeful

Anxious Confident

Sad Happy

Dismissed Well cared for

## Step 6- Remaining pairs

Disagree- agree

Surprised-expected

Frustrated-encouraged

Worried-reassured
