# Supplementary material for: Development and measurement properties of the AxEL (attitude toward education and advice for low-back-pain) questionnaire
Source: Health Qual Life Outcomes. 2022 Jan 10;20:4. doi: 10.1186/s12955-021-01908-4 (PMC8744221; doi:10.1186/s12955-021-01908-4)
Supplement: Supplementary file 2 — Additional file 2. Questionnaire draft. [file 12955_2021_1908_MOESM2_ESM.docx]

# Supplementary Material 2- Preliminary AxEL questionnaire

# 40 items.

## Instructions

We will present 10 statements to you about low back pain. These statements are considered important by experts for the general public to know about low back pain. Following each statement we ask you 4 short questions to evaluate your attitude towards the statement. Choose the most appropriate response.

### When you have back pain, staying active is important. You need to pace yourself to return to your usual activities

| Very frustrating | Very worrying | Strongly disagree | Very surprising |
| --- | --- | --- | --- |
| Frustrating | worrying | Disagree | Surprising |
| Somewhat frustrating | somewhat worrying | Somewhat disagree | Somewhat surprising |
| Neither frustrating nor encouraging | neither worrying nor reassuring | Neither agree nor disagree | Neither surprising nor expected |
| Somewhat encouraging | somewhat reassuring | Somewhat agree | Somewhat expected |
| Encouraging | reassuring | Agree | Expected |
| Very encouraging | very reassuring | Strongly agree | Very much expected |

### When you have low back pain it is important to, take ownership of your own wellbeing

| Very frustrating | Very worrying | Strongly disagree | Very surprising |
| --- | --- | --- | --- |
| Frustrating | worrying | Disagree | Surprising |
| Somewhat frustrating | somewhat worrying | Somewhat disagree | Somewhat surprising |
| Neither frustrating nor encouraging | neither worrying nor reassuring | Neither agree nor disagree | Neither surprising nor expected |
| Somewhat encouraging | somewhat reassuring | Somewhat agree | Somewhat expected |
| Encouraging | reassuring | Agree | Expected |
| Very encouraging | very reassuring | Strongly agree | Very much expected |

### You should see a health practitioner URGENTLY if you have back pain and either of the following: bladder and/or bowel disturbance, significant leg muscle weakness

| Very frustrating | Very worrying | Strongly disagree | Very surprising |
| --- | --- | --- | --- |
| Frustrating | worrying | Disagree | Surprising |
| Somewhat frustrating | somewhat worrying | Somewhat disagree | Somewhat surprising |
| Neither frustrating nor encouraging | neither worrying nor reassuring | Neither agree nor disagree | Neither surprising nor expected |
| Somewhat encouraging | somewhat reassuring | Somewhat agree | Somewhat expected |
| Encouraging | reassuring | Agree | Expected |
| Very encouraging | very reassuring | Strongly agree | Very much expected |

### Most people find that their back pain settles down over a short period of time. If your back pain persists and is worrying you, consult a health professional.

| Very frustrating | Very worrying | Strongly disagree | Very surprising |
| --- | --- | --- | --- |
| Frustrating | worrying | Disagree | Surprising |
| Somewhat frustrating | somewhat worrying | Somewhat disagree | Somewhat surprising |
| Neither frustrating nor encouraging | neither worrying nor reassuring | Neither agree nor disagree | Neither surprising nor expected |
| Somewhat encouraging | somewhat reassuring | Somewhat agree | Somewhat expected |
| Encouraging | reassuring | Agree | Expected |
| Very encouraging | very reassuring | Strongly agree | Very much expected |

### Staying active helps prevent long-term back problems.

| Very frustrating | Very worrying | Strongly disagree | Very surprising |
| --- | --- | --- | --- |
| Frustrating | worrying | Disagree | Surprising |
| Somewhat frustrating | somewhat worrying | Somewhat disagree | Somewhat surprising |
| Neither frustrating nor encouraging | neither worrying nor reassuring | Neither agree nor disagree | Neither surprising nor expected |
| Somewhat encouraging | somewhat reassuring | Somewhat agree | Somewhat expected |
| Encouraging | reassuring | Agree | Expected |
| Very encouraging | very reassuring | Strongly agree | Very much expected |

### Persistent low back pain is influenced by a number of factors - physical, emotional, environmental; so it is important to address each of these areas.

| Very frustrating | Very worrying | Strongly disagree | Very surprising |
| --- | --- | --- | --- |
| Frustrating | worrying | Disagree | Surprising |
| Somewhat frustrating | somewhat worrying | Somewhat disagree | Somewhat surprising |
| Neither frustrating nor encouraging | neither worrying nor reassuring | Neither agree nor disagree | Neither surprising nor expected |
| Somewhat encouraging | somewhat reassuring | Somewhat agree | Somewhat expected |
| Encouraging | reassuring | Agree | Expected |
| Very encouraging | very reassuring | Strongly agree | Very much expected |

### Your pain may not necessarily be related to the extent of damage in your back. Hurt does not necessarily mean harm.

| Very frustrating | Very worrying | Strongly disagree | Very surprising |
| --- | --- | --- | --- |
| Frustrating | worrying | Disagree | Surprising |
| Somewhat frustrating | somewhat worrying | Somewhat disagree | Somewhat surprising |
| Neither frustrating nor encouraging | neither worrying nor reassuring | Neither agree nor disagree | Neither surprising nor expected |
| Somewhat encouraging | somewhat reassuring | Somewhat agree | Somewhat expected |
| Encouraging | reassuring | Agree | Expected |
| Very encouraging | very reassuring | Strongly agree | Very much expected |

### It is not necessary to know the specific cause of your back pain in order to manage the pain effectively.

| Very frustrating | Very worrying | Strongly disagree | Very surprising |
| --- | --- | --- | --- |
| Frustrating | worrying | Disagree | Surprising |
| Somewhat frustrating | somewhat worrying | Somewhat disagree | Somewhat surprising |
| Neither frustrating nor encouraging | neither worrying nor reassuring | Neither agree nor disagree | Neither surprising nor expected |
| Somewhat encouraging | somewhat reassuring | Somewhat agree | Somewhat expected |
| Encouraging | reassuring | Agree | Expected |
| Very encouraging | very reassuring | Strongly agree | Very much expected |

### It is rare for low back pain to be caused by a more serious health problem.

| Very frustrating | Very worrying | Strongly disagree | Very surprising |
| --- | --- | --- | --- |
| Frustrating | worrying | Disagree | Surprising |
| Somewhat frustrating | somewhat worrying | Somewhat disagree | Somewhat surprising |
| Neither frustrating nor encouraging | neither worrying nor reassuring | Neither agree nor disagree | Neither surprising nor expected |
| Somewhat encouraging | somewhat reassuring | Somewhat agree | Somewhat expected |
| Encouraging | reassuring | Agree | Expected |
| Very encouraging | very reassuring | Strongly agree | Very much expected |

### Imaging e.g. x-ray, CT scan or MRI is usually not needed in the majority of cases of low back pain, particularly when you pain has been present for less than 6 weeks. Talk to your doctor about this.

| Very frustrating | Very worrying | Strongly disagree | Very surprising |
| --- | --- | --- | --- |
| Frustrating | worrying | Disagree | Surprising |
| Somewhat frustrating | somewhat worrying | Somewhat disagree | Somewhat surprising |
| Neither frustrating nor encouraging | neither worrying nor reassuring | Neither agree nor disagree | Neither surprising nor expected |
| Somewhat encouraging | somewhat reassuring | Somewhat agree | Somewhat expected |
| Encouraging | reassuring | Agree | Expected |
| Very encouraging | very reassuring | Strongly agree | Very much expected |

## Scoring algorithm

Participants were required to answer every question.

There were ten messages, and four components of attitude, each scored on a 7-point scale (0-6).

For message 1- (0 or 1 or 2 or 3 or 4 or 5 or 6) + (0 or 1 or 2 or 3 or 4 or 5 or 6) + (0 or 1 or 2 or 3 or 4 or 5 or 6) + (0 or 1 or 2 or 3 or 4 or 5 or 6)

For message 2- (0 or 1 or 2 or 3 or 4 or 5 or 6) + (0 or 1 or 2 or 3 or 4 or 5 or 6) + (0 or 1 or 2 or 3 or 4 or 5 or 6) + (0 or 1 or 2 or 3 or 4 or 5 or 6)

Repeat for message 3-10

There could be a maximum score of 24 for each message
