# Supplementary material for: Development and measurement properties of the AxEL (attitude toward education and advice for low-back-pain) questionnaire
Source: Health Qual Life Outcomes. 2022 Jan 10;20:4. doi: 10.1186/s12955-021-01908-4 (PMC8744221; doi:10.1186/s12955-021-01908-4)

# Supplementary Material 3-The Back Beliefs questionnaire and The modified Pain Self Efficacy Questionnaire

## The Back Beliefs Questionnaire (BBQ):

Available form Symonds TL, Burton AK, Tillotson KM, Main CJ. (1996) Do Attitudes and Beliefs Influence Work Loss Due to Low Back Trouble?

Occupational Medicine;46(1):25-32 <https://doi.org/10.1093/occmed/46.1.25>


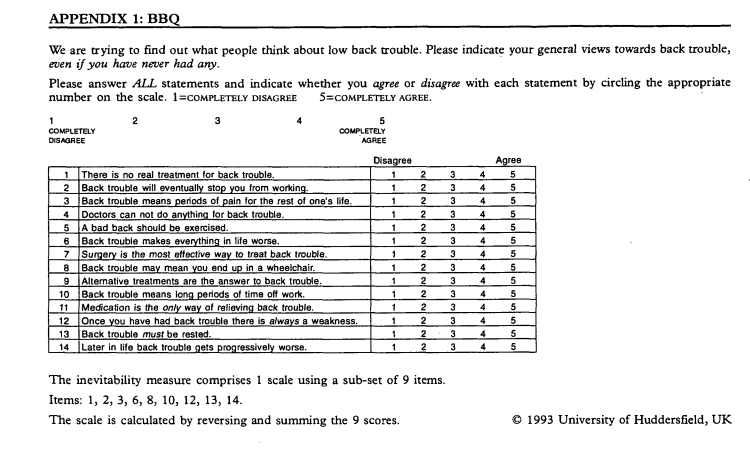


## The modified Pain Self-Efficacy Questionnaire

Available form Nicholas MK, McGuire BE, Asghari A. (2015) A 2-item short form of the Pain Self-efficacy Questionnaire: development and psychometric evaluation of PSEQ-2. *Journal of Pain****;***16(2):153-63 https://doi:10.1016/j.jpain.2014.11.002


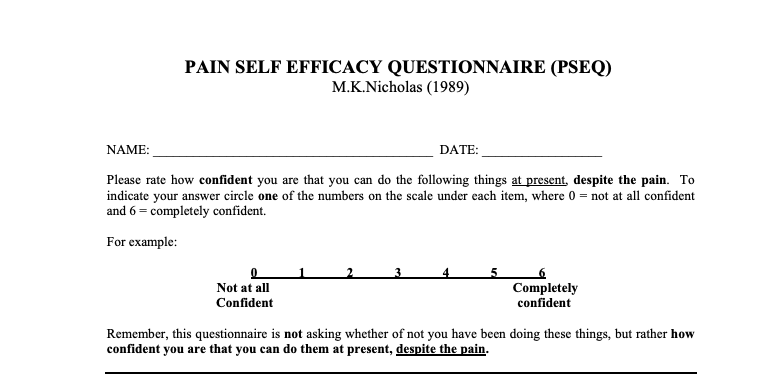

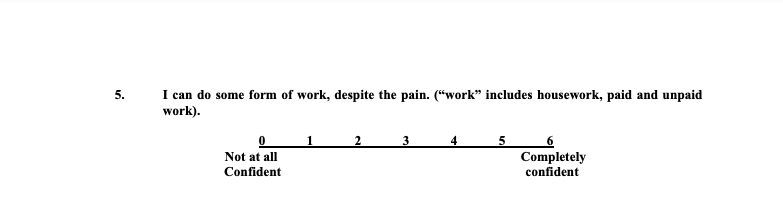

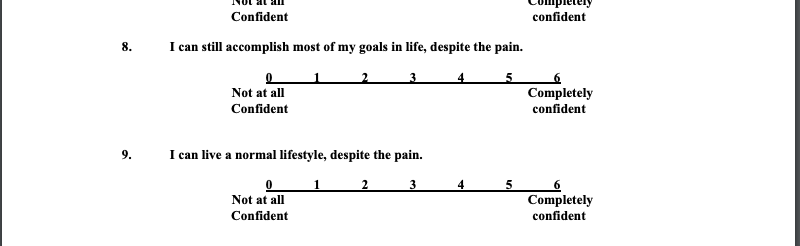

Supplement: Supplementary file 3 — Additional file 3. Back Beliefs Questionnaire & the Modified Pain Self-Efficacy Questionnaire. [file 12955_2021_1908_MOESM3_ESM.docx]
