# Supplementary material for: Development and measurement properties of the AxEL (attitude toward education and advice for low-back-pain) questionnaire
Source: Health Qual Life Outcomes. 2022 Jan 10;20:4. doi: 10.1186/s12955-021-01908-4 (PMC8744221; doi:10.1186/s12955-021-01908-4)
Supplement: Supplementary file 4 — Additional file 4. Exploratory Factor Analysis (EFA) results. [file 12955_2021_1908_MOESM4_ESM.docx]

# Supplementary Material 4- Exploratory Factor Analysis results

| Method | Eigen values | No of items | % Variance | Tucker Lewis Index | Comparative Fit Index (CFI) | Root Mean Square Error of Approximation (RMSEA) | Root Mean Square of the Residual (RMSR) |
| --- | --- | --- | --- | --- | --- | --- | --- |
| Scree Plot | 1 | 26 | 40 | 0.45 | 0.50 | 0.18 | 0.13 |
| **Scree Plot** | **3** | **17** | **65** | **0.77** | **0.84** | **0.15** | **0.06** |
| Theory | 4 | 21 | 61 | 0.74 | 0.82 | 0.13 | 0.06 |
| **Kaiser Criterium (>1)** | **5** | **21** | **69** | **0.84** | **0.91** | **0.11** | **0.04** |
| Kaiser Criterium (>.7) | 7 | 30 | 64 | 0.74 | 0.88 | 0.11 | 0.05 |
| Parallel analysis | 11 | 36 | 75 | 0.91 | 0.82 | 0.06 | 0.02 |
