# Supplementary material for: Development and measurement properties of the AxEL (attitude toward education and advice for low-back-pain) questionnaire
Source: Health Qual Life Outcomes. 2022 Jan 10;20:4. doi: 10.1186/s12955-021-01908-4 (PMC8744221; doi:10.1186/s12955-021-01908-4)
Supplement: Supplementary file 5 — Additional file 5. The three-factor Model. [file 12955_2021_1908_MOESM5_ESM.docx]

# Supplementary Material 5- Exploratory Factor Analysis result

## Step 1- Correlation adequacy?

Bartlett’s test of correlation adequacy [chisq 2928.5, p>0.01].

Mean sampling adequacy (0.9)

## Step 2- Number of Factors

Theory suggests 4; self-management, education, staying active, professional help.

Scree plots suggests three. Scree plot graph included below.

### Appendix 3- Figure 1. Scree plot and parallel analysis


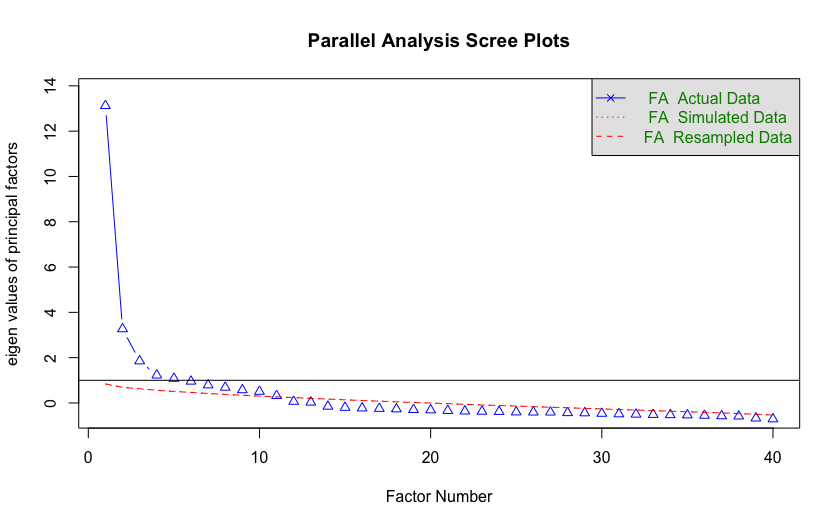


Parallel analysis suggests 11

Kaiser criterion suggests 5.

## Step 3- Simple structure?

Factor rotation-oblique

Fitting estimation- maximum likelihoold

Loading table, correlation factor and item (>.5)

Loading table rounds 1-5 are included below;

### Loading table round 1

| M | L2 M | L1 M | L3 h |  |  |
| --- | --- | --- | --- | --- | --- |
| ea1_agree | 0.1 | 0.61 | 0.03 |  |  |
| ea1_surp | 0.26 | 0.04 | 0.23 | bad | 2 |
| ea1_worry | 0.18 | 0.57 | 0.09 |  |  |
| ea1_frust | 0.18 | 0.63 | 0.08 |  |  |
| ea2_agree | 0.06 | 0.51 | 0.14 |  |  |
| ea2_surp | 0.15 | 0.3 | 0.25 | bad | 6 |
| ea2_worry | -0.05 | 0.66 | 0.21 |  |  |
| ea2_frust | -0.11 | 0.66 | 0.18 |  |  |
| ea3_agree | -0.11 | -0.01 | 0.52 |  |  |
| ea3_surp | -0.02 | -0.05 | 0.57 |  |  |
| ea3_worry | -0.13 | -0.06 | 0.6 |  |  |
| ea3_frust | -0.19 | 0.09 | 0.63 |  |  |
| ea4_agree | 0.1 | -0.07 | 0.62 |  |  |
| ea4_surp | 0.33 | -0.1 | 0.41 | bad | 14 |
| ea4_worry | 0.07 | 0.1 | 0.68 |  |  |
| ea4_frust | 0.02 | 0.09 | 0.69 |  |  |
| ea5_agree | 0.07 | 0.82 | -0.07 |  |  |
| ea5_surp | 0.24 | 0.47 | 0.01 | bad | 18 |
| ea5_worry | 0 | 0.89 | 0 |  |  |
| ea5_frust | -0.03 | 0.91 | -0.04 |  |  |
| ea6_agree | 0.1 | 0.24 | 0.38 | bad | 21 |
| ea6_surp | 0.28 | -0.12 | 0.46 | bad | 22 |
| ea6_worry | 0.12 | 0.21 | 0.45 | bad | 23 |
| ea6_frust | 0.02 | 0.24 | 0.46 | bad | 24 |
| ea7_agree | 0.58 | 0.15 | 0.1 |  |  |
| ea7_surp | 0.59 | -0.08 | 0.13 |  |  |
| ea7_worry | 0.57 | 0.24 | 0.04 |  |  |
| ea7_frust | 0.51 | 0.25 | 0.11 |  |  |
| ea8_agree | 0.83 | -0.02 | -0.12 |  |  |
| ea8_surp | 0.73 | -0.23 | 0.02 |  |  |
| ea8_worry | 0.83 | 0.05 | -0.12 |  |  |
| ea8_frust | 0.81 | 0.02 | -0.04 |  |  |
| ea9_agree | 0.67 | 0.04 | 0.07 |  |  |
| ea9_surp | 0.64 | -0.14 | 0.13 |  |  |
| ea9_worry | 0.62 | 0.19 | 0 |  |  |
| ea9_frust | 0.57 | 0.23 | -0.01 |  |  |
| ea10_agree | 0.51 | 0.14 | 0.14 |  |  |
| ea10_surp | 0.57 | -0.05 | 0.16 |  |  |
| ea10_worry | 0.48 | 0.26 | 0.11 | bad | 39 |
| ea10_frust | 0.45 | 0.21 | 0.17 | bad | 40 |
|  |  |  |  |  |  |

### Loading table round 2

|  |  |  |  |  |  |
| --- | --- | --- | --- | --- | --- |
|  | ML2 | ML1 | ML3 |  |  |
| ea1_agree | 0.07 | 0.65 | 0 |  |  |
| ea1_worry | 0.16 | 0.59 | 0.1 |  |  |
| ea1_frust | 0.16 | 0.64 | 0.11 |  |  |
| ea2_agree | 0.04 | 0.58 | 0.04 |  |  |
| ea2_worry | -0.04 | 0.69 | 0.13 |  |  |
| ea2_frust | -0.1 | 0.67 | 0.14 |  |  |
| ea3_agree | -0.1 | 0.08 | 0.35 | bad | 9 |
| ea3_surp | -0.03 | 0.1 | 0.32 | bad | 10 |
| ea3_worry | -0.1 | 0.02 | 0.43 | bad | 11 |
| ea3_frust | -0.18 | 0.16 | 0.49 | bad | 12 |
| ea4_agree | 0.06 | -0.1 | 0.71 |  |  |
| ea4_worry | 0.04 | 0.02 | 0.87 |  |  |
| ea4_frust | -0.01 | 0 | 0.9 |  |  |
| ea5_agree | 0.05 | 0.84 | -0.09 |  |  |
| ea5_worry | -0.01 | 0.88 | 0 |  |  |
| ea5_frust | -0.04 | 0.89 | -0.02 |  |  |
| ea7_agree | 0.52 | 0.23 | 0.09 |  |  |
| ea7_surp | 0.52 | 0.01 | 0.07 |  |  |
| ea7_worry | 0.52 | 0.28 | 0.04 |  |  |
| ea7_frust | 0.47 | 0.29 | 0.12 | bad | 28 |
| ea8_agree | 0.87 | -0.06 | -0.05 |  |  |
| ea8_surp | 0.75 | -0.23 | 0.04 |  |  |
| ea8_worry | 0.88 | 0.02 | -0.05 |  |  |
| ea8_frust | 0.86 | -0.01 | 0.02 |  |  |
| ea9_agree | 0.61 | 0.11 | 0.06 |  |  |
| ea9_surp | 0.56 | -0.03 | 0.07 |  |  |
| ea9_worry | 0.55 | 0.25 | 0.01 |  |  |
| ea9_frust | 0.52 | 0.29 | 0 |  |  |
| ea10_agree | 0.42 | 0.2 | 0.12 | bad | 37 |
| ea10_surp | 0.47 | 0.05 | 0.06 | bad | 38 |
|  |  |  |  |  |  |

### Loading table round 3

| ML | 2 ML | 3 ML | 1 h2 |  |  |
| --- | --- | --- | --- | --- | --- |
| ea1_agree | 0.67 | 0.04 | 0.01 |  |  |
| ea1_worry | 0.65 | 0.08 | 0.11 |  |  |
| ea1_frust | 0.7 | 0.09 | 0.09 |  |  |
| ea2_agree | 0.63 | 0 | 0.01 |  |  |
| ea2_worry | 0.77 | -0.03 | -0.02 |  |  |
| ea2_frust | 0.78 | -0.04 | -0.1 |  |  |
| ea4_agree | 0.31 | -0.02 | 0.08 | bad | 13 |
| ea4_worry | 0.51 | 0 | 0.03 |  |  |
| ea4_frust | 0.51 | -0.01 | 0 |  |  |
| ea5_agree | 0.78 | 0.02 | 0.03 |  |  |
| ea5_worry | 0.87 | -0.01 | -0.01 |  |  |
| ea5_frust | 0.87 | -0.03 | -0.02 |  |  |
| ea7_agree | 0.27 | 0.26 | 0.25 | bad | 25 |
| ea7_surp | 0.08 | 0.33 | 0.15 | bad | 26 |
| ea7_worry | 0.3 | 0.29 | 0.24 | bad | 27 |
| ea8_agree | -0.02 | 0.85 | 0.04 |  |  |
| ea8_surp | -0.09 | 0.8 | -0.12 |  |  |
| ea8_worry | 0.04 | 0.89 | 0.04 |  |  |
| ea8_frust | 0.06 | 0.88 | 0.01 |  |  |
| ea9_agree | -0.02 | 0.11 | 0.75 |  |  |
| ea9_surp | -0.12 | 0.1 | 0.66 |  |  |
| ea9_worry | 0.01 | -0.06 | 0.99 |  |  |
| ea9_frust | 0.06 | -0.01 | 0.88 |  |  |
|  |  |  |  |  |  |

### Loading table round 4

| ML2 | ML3 | ML1 | h2 |  |  |
| --- | --- | --- | --- | --- | --- |
| ea1_agree | 0.66 | 0.03 | 0.01 |  |  |
| ea1_worry | 0.64 | 0.08 | 0.12 |  |  |
| ea1_frust | 0.7 | 0.09 | 0.09 |  |  |
| ea2_agree | 0.62 | 0 | 0.02 |  |  |
| ea2_worry | 0.78 | -0.02 | -0.02 |  |  |
| ea2_frust | 0.78 | -0.03 | -0.1 |  |  |
| ea4_worry | 0.49 | 0.01 | 0.04 | bad | 15 |
| ea4_frust | 0.49 | 0 | 0.01 | bad | 16 |
| ea5_agree | 0.78 | 0.02 | 0.03 |  |  |
| ea5_worry | 0.87 | -0.01 | -0.01 |  |  |
| ea5_frust | 0.87 | -0.02 | -0.02 |  |  |
| ea8_agree | -0.02 | 0.84 | 0.05 |  |  |
| ea8_surp | -0.09 | 0.78 | -0.12 |  |  |
| ea8_worry | 0.04 | 0.9 | 0.04 |  |  |
| ea8_frust | 0.06 | 0.89 | 0.01 |  |  |
| ea9_agree | -0.02 | 0.12 | 0.74 |  |  |
| ea9_surp | -0.12 | 0.09 | 0.65 |  |  |
| ea9_worry | 0.01 | -0.05 | 0.99 |  |  |
| ea9_frust | 0.07 | 0 | 0.87 |  |  |

### Loading table round 5

| ML | 2 ML | 3 ML | 1 h2 |  |  |  |
| --- | --- | --- | --- | --- | --- | --- |
| ea1_agree | 0.66 | 0.03 | 0.01 | 1 |  |  |
| ea1_worry | 0.63 | 0.09 | 0.12 | 3 |  |  |
| ea1_frust | 0.68 | 0.09 | 0.1 | 4 |  |  |
| ea2_agree | 0.61 | 0 | 0.02 | 5 |  |  |
| ea2_worry | 0.76 | -0.02 | -0.01 | 7 |  |  |
| ea2_frust | 0.77 | -0.02 | -0.09 | 8 |  |  |
| ea5_agree | 0.8 | 0.01 | 0.03 | 17 |  |  |
| ea5_worry | 0.88 | -0.01 | -0.01 | 19 |  |  |
| ea5_frust | 0.88 | -0.03 | -0.02 | 20 |  |  |
| ea8_agree | -0.01 | 0.84 | 0.05 |  | 29 |  |
| ea8_surp | -0.09 | 0.78 | -0.11 |  | 30 |  |
| ea8_worry | 0.05 | 0.9 | 0.04 |  | 31 |  |
| ea8_frust | 0.06 | 0.89 | 0.01 |  | 32 |  |
| ea9_agree | -0.02 | 0.12 | 0.74 |  |  | 33 |
| ea9_surp | -0.13 | 0.09 | 0.65 |  |  | 34 |
| ea9_worry | 0.01 | -0.05 | 0.99 |  |  | 35 |
| ea9_frust | 0.07 | -0.01 | 0.87 |  |  | 36 |

## Step 4- Fit statistics

### Goodness of fit

1. Non-normed Fit index/ Tucker Lewis index =0.77

2. Comparative Fit index =0.84

### Residual statistics

1.Root mean square error of approximation (RMSEA) =0.15

2. Root mean square of the residual (RMSR) =0.06

## Step 5-Labelling

Factor 1 Attitude toward staying active

Factor 2 Attitude toward benign nature of low back pain

Factor 3 Attitude toward absence of serious pathology
